# Antimicrobial residues and compositional quality of informally marketed raw cow milk, Lamu West Sub-County, Kenya, 2015

**DOI:** 10.11604/pamj.supp.2017.28.1.9279

**Published:** 2017-11-03

**Authors:** George Kiage Ondieki, Jackson Nyarongi Ombui, Mark Obonyo, Zeinab Gura, Jane Githuku, Austine Bitek Orinde, Joseph Kangangi Gikunju

**Affiliations:** 1 Field Epidemiology and Laboratory Training Program Kenya (K-FELTP), Kenya,; 2 Jomo Kenyatta University of Agriculture and Technology, Institute of Tropical Medicine and Infectious Diseases (ITROMID), Department of Medical Laboratory Science, Kenya,; 3 Department of Public Health, Pharmacology and Toxicology, Faculty of Veterinary Medicine, University of Nairobi, Kenya,; 4 Zoonotic Disease Unit, Ministry of Agriculture, Livestock Development and Fisheries, Kenya

**Keywords:** Antimicrobial residues, composition, adulteration, milk, Kenya

## Abstract

**Introduction:**

unadulterated milk, free of antimicrobial residues is important for industrial processing and consumers’ health. Antimicrobial residues in foods of animal origin can cause adverse public health effects like drug resistance and hypersensitivity. Milk produced in Lamu West sub-county is sold raw directly to consumers. We estimated the compositional quality and prevalence of antimicrobial residues in informally marketed raw cow milk in Lamu West Sub-County, Kenya.

**Methods:**

we randomly recruited 152 vendors and 207 farmers from four randomly selected urban centers in a cross-sectional study and interviewed them using a pretested standardized questionnaire. A100-ml raw milk sample was aseptically collected from each vendor and farm and tested for antimicrobial residues using Charm Blue Yellow II kit following the European Union Maximum Residue Limits (EU-MRLs) while an Ekomilk® Analyzer was used to measure compositional quality where samples with either solid not fat (SNF) < 8.5 or added water ≥ 0.01% or both were considered adulterated. We analyzed data using univariate analysis and unconditional logistic regression to calculate odds ratios (OR) and 95% confidence intervals (CI).

**Results:**

thirty-two of the 207 (15.5%) samples from farmers and 28 (18.4%) of the 152 samples from vendors tested positive for antimicrobial residues. Thirty-six (17.4 %) samples from farmers and 38 (25.0%) from vendors were found to be adulterated with water. Farmers’ awareness of the danger of consuming milk with antimicrobial residues and farmers having training on good milking practices were protective against selling milk with antimicrobial residues (adjusted OR and 95% CI 0.20, 0.07-0.55 and 0.33, 0.11-0.99, respectively).

**Conclusion:**

the antimicrobial residues above EU MRLs and adulteration of raw marketed cow milk observed in this study provide evidence for routine testing of marketed milk and educating farmers to observe antimicrobial withdrawal period.

## Introduction

Unadulterated high quality milk that is free of antimicrobial residues is of interest to farmers, consumers and milk processing companies. Such milk enables farmers to get a fair price for their produce while processors are assured of a raw material suitable for manufacture of various dairy products. Consumers are also guaranteed of getting a healthy product at a good value [1].

Compositional quality of milk is determined by measuring its constituents and physico-chemical properties including: added water, butter fat, solid non-fat (SNF), protein, specific gravity and freezing point [2]. Adulteration of milk refers to the alteration of the natural composition of milk by extraction of one or more of its components (such as butter fat) or addition of some substances (such as water). Milk adulteration by addition of substances such as water interferes with the hygienic, compositional, nutritional and processing qualities of the milk, while extraction of components from milk lowers the value for money paid by consumers or processors [3].

Although antimicrobials are useful for treatment of infections, their occurrence in foods of animal origin as residues can cause adverse public health effects such as drug resistance [4,5] and hypersensitivity caused by penicillins and sulphonamides antibiotic groups [6,7]. Their occurrence in milk also causes huge economic losses in milk processing industries by interfering with the manufacture of cultured products such as yoghurt and cheese through inhibition of starters and rejection of milk from farms that test positive for antimicrobials [8]. Antibiotics used in veterinary practice are identical or closely related to those used in human medicine. Hence, any improper use or exposure in either can easily result in cross-resistance [5].

To protect the public against possible health risks caused by antimicrobial residues and consumption of milk of unacceptable compositional quality, regulations have been developed both locally and internationally to ensure observance of withdrawal periods after antimicrobial therapy and proper handling and marketing of milk. International regulations include European Union Maximum Residual Limits (EU MRLs) and the Codex Alimentarious Commission (CAC) [9,10]. In Kenya, quality and safety of milk is regulated by the Dairy Industry Act [11], Public Health Act [12] and the Standards Act [13]. However, such regulations might not be adhered to or enforced, as is the case in many developing countries [14].

The dairy industry in Lamu County is in its early stages of development. As of 2015, all the milk produced in Lamu West sub-county was sold raw directly to consumers without undergoing any quality assessment and safety assurance against presence of antimicrobial residues. No investigations have been carried out to assess the extent and nature of the risks consumers of marketed raw cow milk in Lamu County may be exposed to. This study assessed the compositional quality of milk and estimated the prevalence of antimicrobial residues in informally marketed raw cow milk in Lamu West Sub-County with the goal of providing feedback to farmers, vendors, consumers, policy makers and enforcers.

## Methods

### Study area and design

**Figure 1:**
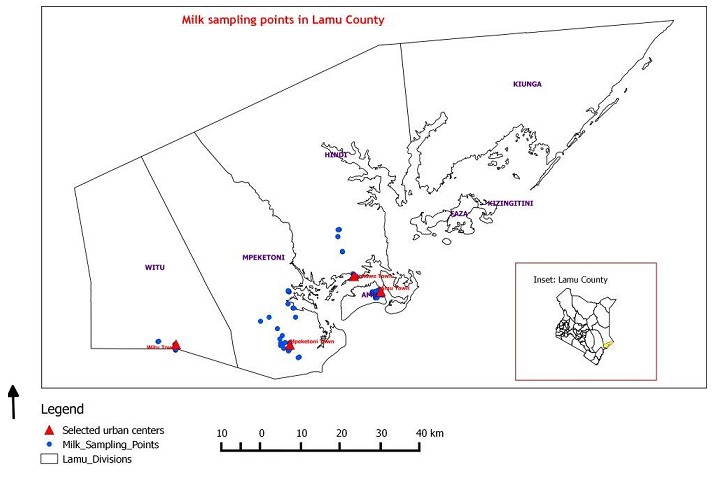
a map showing the study site, Lamu West Sub-County, the selected urban centres

A cross-sectional study was conducted in Lamu West Sub County of Lamu County, in the northern coastal region of Kenya during the months of July to November 2015. Lamu County is made up of two sub-counties: Lamu East and Lamu West. Lamu West Sub-County is made up of four administrative divisions, namely: Amu, Hindi, Mpeketoni and Witu; and six urban centres, namely: Amu, Mokowe, Hindi, Mpeketoni, Kibaoni and Witu, of which Amu is an island in the Indian Ocean (Figure 1).

Lamu West Sub-County has a population of 80,000 persons. The main economic activities in this region are fishing, tourism, livestock and crop farming. The livestock species kept here are mainly cattle, sheep goats, donkeys and poultry. Lamu West has an estimated cattle population of 126,250. The cattlerearing systems practiced here are: pastoralism, semi-zero grazing and zero grazing. Pastoralism, practiced in Hindi, Mpeketoni and Witu divisions, entails grazing large herds of local breeds of cattle (Boran and Zebu) in public or communally owned land and occasionally migrating to neighboring counties in search of greener pastures and drinking water. Semi-zero grazing, practiced in Hindi, Mpeketoni and Amu divisions, entails grazing cattle in the fields and providing supplementary feeding with fodder or commercial feeds. The breeds of cattle kept in this type of farming are cross-breeds and exotic breeds (Jersey, Guernsey, and Friesian). In zero grazing, practiced in Amu division, cattle are kept in enclosures and rely of fodder and commercial feeds. The types of cattle kept here are cross-breeds and exotic breeds. In Lamu County the milk from these cattle is sold raw to consumers who then boil it before consumption. The only link between the producer (farmer) and the consumer are small scale traders or milk vendors.

### Sample size calculation and sampling procedure

Sample sizes for vendors and farmers were determined separately using the Cochran formula of 1977, using estimated prevalence of antimicrobial residues of 16% for milk from farms and 11.1% for milk from vendors [8]. We assumed Z-value for 95% confidence level as 1.96, and the precision (margin of error) at 5%. A total of 152 milk vendors and 207 livestock farmers were estimated as the sample size needed to achieve power of 80%.

Four of the six urban centres in the sub county were randomly selected for this study. These urban centres were: Amu, Mokowe, Mpeketoni and Witu. A preliminary visit was made to the selected market centres and with the help of key informants (the Public Health Officers, Livestock Production Officers, Veterinary Officers, Local Authority Trade Officers, milk marketing groups or associations and milk selling points), a sampling frame of informal raw milk vendors was created for each selected urban centre. A milk vendor was defined as any person who obtained milk from own farm or bought milk from one or more farms or milk outlets and sold it by hawking along the pathways or at raw milk selling shops within the milk supply catchment of the selected urban centers. Using the same key informants, another sampling frame was created comprising of livestock farmers, where a legible livestock farmer was defined as any person with lactating cattle within the milk supply catchment area of each selected urban centre and offered milk for sale at their farms. The established sampling frames were made of two groups: 784 livestock farmers with lactating cattle and 251 vendors. The number of farmers and vendors sampled from each centre were determined proportionate to number of farmers and vendors in each selected centre. Sampling units were individual vendors and farmers. Those who participated in the study were randomly selected using simple random sampling. Those who refused to consent or participate in the study were replaced from the sampling frame using simple random sampling.

### Milk specimen and data collection

After obtaining consent and appropriately compensating the farmers and vendors for the milk, 100 ml milk specimens were aseptically collected in sterile bottles from each respondent, stored in ice-packed cool boxes and transported to Lamu County hospital laboratory where they were analyzed on the same day they arrived. A pretested structured questionnaire translated into Kiswahili was administered to each vendor and farmer to collect data on demographics and practices that might affect the compositional quality of the milk and occurrence of antimicrobial residues such as livestock treatment practices, observance of milk withdrawal period following antibiotic therapy, training in good milking and milk handling practices, practices used to prolong freshness of milk and methods used by vendors in selling milk.

### Compositional quality

The milk specimens from vendors and from farmers were analyzed for compositional quality using Ekomilk® Ultrasonic Milk Analyzer (EON Trading LLC USA), as per manufacturer’s instructions and as similarly done by Kunda et al (2015) [15]. The milk specimen vials were brought out of the cool box and allowed to thaw to room temperature. Each milk specimen vial was shaken gently to thoroughly mix the contents after which 20 ml of the milk specimen was transferred into the analyzer cup. The cup was placed below the aspiration tube of the Ekomilk® Ultrasonic Milk Analyzer and connected to power to start the analysis. The parameters estimated by the analyzer were: added water, butter fat, solid non-fat (SNF), protein percentage, specific gravity and freezing point. Adulterated milk was identified using standard values [2,15] by having SNF < 8.5%, added water ≥ 0.01%, specific gravity outside the normal range (1.026-1.036 Kg/l, butterfat < 3.3% or > 7.0%;and freezing point outside the normal range of between -0.525°C and -0.565°C.

### Testing for antimicrobial residues

Charm Blue Yellow II kit was used to test for presence of antimicrobial residues using a standard method as described by the manufacturer [16]. A 100-ml milk specimen obtained from Witu Veterinary Farm from a cow that had not been exposed to antibiotics therapeutically, prophylactically or as feed additives for the past 12 months was used as a negative control and was confirmed negative by the Charm Blue Yellow II test kit. A positive control was prepared by reconstituting the provided 4 parts per billion (ppb) Penicillin G Standard with 10.0 ml of a negative control, shaken and allowed to stand refrigerated for 15 minutes. The controls were put in 0.5 ml aliquots in clean vials and frozen at -15°C for later use. Whenever the controls were needed, they were slowly thawed overnight in a refrigerator and shaken well before use.

Each milk specimen (or control) was shaken and 50 µl was pipetted into the purple agar portion of the well. A clear sealing tape was applied and pressed firmly to seal the rim of each well to prevent them from drying. The prepared wells were put into an air incubator at 64 ± 1°C for 2 hours 55 minutes. After incubation, the wells were removed from the air incubator, allowed to settle for 5 minutes on the desk, for colour development. Colour observation was done in comparison with the reference colours provided by the manufacturer [16]. Yellow or yellow/green wells were interpreted as negative, whereas blue/purple wells were interpreted as positive. Grey coloured wells, (referred to as ‘Caution’ by the manufacturer) were interpreted as positive. From the initial positive results, 600 µl of milk was heated in a test tube to boiling point for 3 minutes. Then they were allowed to cool to room temperature and shaken. The heated specimens were run in duplicate along with a negative and positive control and unheated milk specimen in the same procedure as above. Specimens which tested positive after heat treatment were interpreted as ‘Blue Yellow II Test positive’ hence contained antibiotics. Specimens that tested negative after heat treatment were considered to contain a non-antibiotic heat sensitive inhibitor.

### Statistical analysis

The data was entered, cleaned and analyzed in Ms Excel™ 2007 and EPI Info 7™. In univariate analyses, proportions were calculated for categorical variables and means and medians for continuous variables. Bivariate analysis (Pearson chi square and Fischer’s exact tests) was carried out to examine the association between the presence of antimicrobial residues or compositional quality of raw marketed milk and other factors with factors with p-value ≤ 0.05 considered statistically significant. Odds ratios (OR) and 95% confidence intervals (CI) were calculated. Factors in bivariate analyses with p-value ≤ 0.1 were included in a forward selection unconditional logistic regression model to control for confounders and identify independent factors associated with the occurrence of antimicrobial residues in milk and milk adulteration as identified by adjusted odds ratios (AORs) and 95% CIs. Factors with p-value < 0.05 in the final model were considered significant. Comparison of proportions was made using a 2-sample z-test with two tailed comparisons at 0.05 level of significance. Analysis for antimicrobial residue was not done for vendors since the assumption was that majority of antimicrobial residue occurred at the farm level due to lack of observance of antimicrobial withdrawal periods by farmers; and vendors had no role in occurrence of antimicrobial residues in milk as much as the study found antimicrobial residues in milk marketed by vendors.

### Ethical clearance

The aim and procedures of the study were explained to the study participants who were required to give written informed consent prior to their voluntary participation in the study. Milk specimens were collected from only those who consented and the specimens were only used to assess quality characteristics and antimicrobial residues. Confidentiality of laboratory information and data was observed and maintained through password protected computers and observing good professional conduct. Ethical clearance and approval for this study was obtained from Jaramogi Oginga Odinga Teaching and Referral Hospital Ethical Review Committee, Ref. ERC.1B/VOL.1/158. Approval was also obtained from the Board of post graduate studies of Jomo Kenyatta University of Agriculture and Technology (JKUAT), the Lamu County Veterinary Officer and the Lamu County Director of Health, to use the institution’s laboratory facility.

## Results

### Socio-demographic characteristics of respondents

Only three vendors and two farmers declined to participate in the study due to lack of time to respond to the questionnaire, and were replaced by randomly sampling again from the established sampling frames. One hundred and fifty two vendors and 207 farmers were enrolled into the study from Amu, Mokowe, Mpeketoni and Witu urban centers of Lamu West Sub-County. The socio-demographic characteristics of the study participants varied by age, sex, level of education, type of livestock production system, and mode of milk vending business (Table 1).

**Table 1:** socio-demographic characteristics of study participants in Lamu West Sub County, 2015

Characteristics	Total Farmers (N=207) n (%)	Total Vendors (N=152) n (%)
**Selected Market Centres**		
Amu	27 (13.0)	46 (30.3)
Mokowe	40 (19.3)	39 (25.7)
Mpeketoni	52 (25.1)	44 (28.9)
Witu	88 (42.6)	23 (15.1)
**Sex**		
Female	44 (21.3)	101 (67.1)
Male	163 (78.7)	51 (33.5)
**Age groups (years)**		
<45 (19-44)	93 (44.9)	102 (67.1)
≥ 45 (45-80)	114 (55.1)	50 (32.9)
**Livestock production System**		
Pastoralism	157 (75.9)	-
Non pastoralism	50 (24.1)	-
**Level of education**		
< Secondary Education	130 (62.8)	114 (75.0)
≥ Secondary Education	77(37.2)	38 (25.0)
**Type of vendor**		
Kiosk-Shop	-	15 (9.9)
Hawker	-	137 (90.1)
**Who treats their livestock**		
Self	93 (44.9)	-
Veterinarian	114 (55.1)	-

### Compositional quality of milk

The median butterfat content of the marketed raw milk from farms was 5.21 (range 2.02-9.47) whereas that from vendors was 5.25(range 2.26–9.34).An acceptable range of butterfat (3.3-7.0%) was observed in the raw milk from 92.7% (192/207) of farmers and 92.1% (140/152) of vendors. Unacceptable values of SNF, specific gravity, added water and freezing points were also observed in samples from both farmers and vendors (Table 2). Overall, 82.6% (95% CI: 77.0-87.3) of marketed raw cow milk from farms and 75.0% (95% CI 66.7-81.4) from vendors were of acceptable compositional quality.

**Table 2:** compositional quality of informally marketed raw cow milk, Lamu West, 2015

	Farmers, N=207		Vendors, N=152		
Milk Component	Median (Range)	No. out of normal range	Median (Range)	No. out of normal range	Normal Range
Butter Fat %	5.21 (2.02-9.47)	15/207	5.25 (2.26-9.34)	12/152	3.3-7.0
SNF	9.32 (5.86-12.1)	36/207	9.29 (5.45-12.4)	38/152	> 8.5-12.0
Specific Gravity kg/l	1.030 (1.018-1.037)	36/207	1.029 (1.018-1.037)	38/152	1.026-1.036
Added water %	14.28 (0.37-27.90)	36/207	14.04 (0.48-27.6)	38/152	0.00
Freezing point (0C)	-0.597 (-0.401- -0.733)	36/207	-0.595 (-0.402 –0.649)	38/152	-0.525 - -0.565
Protein %	3.53 (2.26-5.27)	5/207	3.52 (2.30- 4.68)	3/152	2.9-5.0

### Prevalence of antimicrobial residues and poor compositional quality

Overall, 15.5% (95% CI: 11.0-20.9) of the samples from farmers and 18.4% (95% CI: 12.9-25.2) of the samples from vendors were found to have antimicrobial residues above the EU MRLs (p-value = 0.467). A significant difference between the prevalence of antimicrobial residues in milk sold by farmers compared to that sold by vendors was only observed in Witu (12.5% vs 30.4%; p = 0.038) (Table 3).

**Table 3:** prevalence of antimicrobial residues in informally marketed raw cow milk per urban centre from farmers and vendors, Lamu West Sub-County, 2015

Urban centre	No. of milk samples		Antimicrobial residues		
	From farmers	From vendors	Positive milk samples from farmers n (%)	Positive milk samples from vendors n (%)	p-value
Amu	27	46	6(22.2)	8(17.4)	0.615
Mokowe	40	39	7(17.5)	5(12.8)	0.560
Mpeketoni	52	44	8(15.3)	8(18.2)	0.704
Witu	88	23	11(12.5)	7(30.4)	**0.038**
**Total**	**207**	**152**	**32(15.5)**	**28(18.4)**	0.467

From the interview findings, 28.5% (59/207) of the farmers and 8.6% (13/152) of the vendors acknowledged to be using a herbal substance with a local name “mpingo” which they applied by smoking the inner side of wooden milk handling containers, to serve as a milk preservative. On laboratory analysis using Charm Blue Yellow test, 20% (41/207) of the milk samples from farmers and 5.9% (9/152) of samples from vendors indicated the presence of a non-antibiotic heat-sensitive inhibitor. Of the 41 positive milk samples from farmers, 63.4% (26/41) were from Witu and 21.9% (9/41) from Mpeketoni. Of the milk samples from farmers, 17.4% (95%CI: 12.7-23.0) and 25.0% (95%CI: 20.6-36.6) from vendors were found to be of poor compositional quality, adulterated by addition of water (p = 0.786). A difference was observed between compositional quality of milk sold by farmers and vendors in Amu (7.4% vs 47.8%; p < 0.001) and Mokowe (27.5% vs 7.7%; p = 0.021) respectively (Table 4).

**Table 4:** prevalence of poor compositional quality of informally marketed raw cow milk per urban centre from farmers and vendors, Lamu West Sub-County, 2015

Urban centre	No. of milk samples		Poor compositional quality		
	From farmers	From vendors	Poor compositional quality milk samples from farmers n (%)	Poor compositional quality milk samples from vendors n (%)	p-value
Amu	27	46	2(7.4)	22(47.8)	**< 0.001**
Mokowe	40	39	11(27.5)	3(7.7)	**0.021**
Mpeketoni	52	44	10(19.2)	8 (18.2)	0.900
Witu	88	23	13(14.8)	5(21.7)	0.424
**Total**	**207**	**152**	**36(17.4)**	**38(25.0)**	0.786

### Comparison between compositional quality and prevalence of antimicrobial residues in milk marketed by farmers and vendors

Overall, 70.5% (95% CI: 64.1-76.4) samples from farmers and 63.2% (95%CI: 55.3-70.6) from vendors were both of good compositional quality and free of antimicrobial residues. However 3.4% (95%CI: 1.5-6.6) of milk samples from farmers and 6.6% (95%CI 3.4-11.4) from vendors contained antimicrobial residues and were of poor compositional quality (p = 0.159).

### Factors associated with presence of antimicrobial residues in marketed raw cow milk among farmers

Farmers who had less than secondary level of education were three times more likely to sell milk with antimicrobial residues (OR 2.98, 95% CI: 1.16-7.56) compared to farmers who had secondary level of education and above. Farmers who were aware of dangers of consuming milk with antimicrobial residues were less likely to sell milk with antimicrobial residues compared to those farmers who were not aware (OR 0.20, 95% CI: 0.07-0.55). Those farmers who had some training on good milking practices were less likely to sell milk with antimicrobial residues compared to those farmers who did not have any training on good milking practices (OR 0.32; 95% CI: 0.11-0.96). Farmers’ awareness of dangers of consuming milk with antimicrobial residues and farmers’ training on good milking practices were retained as independent factors protective against selling milk with antimicrobial residues (Table 5).

**Table 5:** factors associated with presence of antimicrobial residues in informally marketed raw cow milk by farmers in Lamu West Sub County, 2015

Characteristics	Residues Positive n (%)	Residues Negative n (%)	Crude OR (95%CI)	p-value	Adjusted OR (95%CI)	p-value
**Age Group**						
≥ 45 years (45-80)	20 (17.5)	94 (82.5)	1.43 (0.66-3.12)	0.440	-	-
< 45 years (19-44)	12 (12.9)	181(87.1)				
**Level of education**						
< Secondary Education	26 (20.0)	104(80.0)	**2.98 (1.16-7.56)**	**0.027**	1.49(0.52-4.31)	0.461
≥ Secondary Education	6 (7.8)	71 (92.2)	1.00			
**Who treats your sick livestock**						
Veterinarian	14 (12.3)	100 (87.7)	0.58 (0.27-1.25)	0.180	-	-
Other	18 (19.4)	75 (80.6)	1.00			
**Sex**						
Male	22(13.5)	141(86.5)	0.53(0.23-1.22)	0.158	-	-
Female	10(22.7)	34(77.3)	1.00			
**Aware of danger of the residues**						
Aware of danger	5 (5.6)	84(94.4)	**0.20(0.07-0.55)**	**<0.001**	**0.20(0.07-0.55)**	**0.002**
Not aware of danger	27 (22.9)	91(77.1)	1.00			
**Livestock Production System**						
Pastoralist	23 (14.6)	134 (85.4)	0.78 (0.33-1.82)	0.653	-	-
Non-Pastoralist	9 (18)	41 (82)	1.00			
**Training on good milking practices**						
Trained	4 (6.9)	54 (93.1)	**0.32(0.11-0.96)**	**0.033**	**0.33(0.11-0.99)**	**0.048**
Not trained	9 (18.8)	121 (81.2)	1.00			

### Factors associated with poor compositional quality of marketed raw cow milk among farmers and vendors

Farmers who had at least secondary level of education were three times more likely to market milk of poor compositional quality (OR 2.88, 95% CI: 1.38-5.99) compared to those with primary level of education or no formal education. Pastoralist farmers were three times more likely to sell milk of poor compositional quality (OR 2.94, 95% CI: 0.99-8.78) as compared to non-pastoralist farmers. Of the 32 pastoralist farmers found selling milk of poor compositional quality 19 (59.4%) had attained at least secondary level of education. Adjusting for factors simultaneously, farmers having secondary level of education and above (AOR 3.03, 95% CI: 1.44-6.39) and being a pastoralist farmer (AOR 3.20, 95% CI: 1.05-9.71) were retained as independent risk factors against marketing of milk of poor compositional quality (Table 6).

**Table 6:** factors associated with poor compositional quality of informally marketed raw cow milk by farmers in Lamu West Sub County, 2015

Characteristics	Poor Quality n (%)	Good Quality n (%)	Crude OR (95%CI)	p-value	Adjusted OR (95%)	p-value
**Age Group**						
≥ 45 years (45-80)	18 (19.4)	75 (80.6)	1.28 (0.62-2.63)	0.581	-	-
< 45 years (19-44)	18 (15.8)	96 (84.2)	1.00			
**Level of education**						
≥ Secondary Education	21 (27.3)	56 (56)	**2.88 (1.38-5.99)**	**0.007**	**3.03 (1.44-6.39)**	**0.004**
< Secondary Education	15 (11.5)	115 (88.5)	1.0	-	-	-
**Sex**						
Male	27 (16.6)	136 (83.4)	0.77 (0.33-1.79)	0.511	-	-
Female	9 (20.5)	35(79.5)	1.00			
**Training on good milking practices**						
Trained	10 (17.2)	48 (82.8)	0.84 (0.40-1.99)	0.842	-	-
Not trained	26 (19.0)	111 (81.0)	1.00			
**Livestock Production System**						
Pastoralist	32 (20.4)	125(79.6)	2.94 (0.99-8.78)	**0.053**	**3.20 (1.05-9.71)**	**0.040**
Non-Pastoralist	4(8)	46 (92)				

Male vendors were three times more likely to market milk of poor compositional quality (OR 3.46, 95% CI: 1.61-7.47) compared to female vendors. Vendors who had been trained on good milk handling practices were more likely to market milk of poor compositional quality (OR 17.12 CI: 1.93-151.7). Being a male vendor was retained as the only independent risk factor associated with marketing of milk of poor compositional quality amongst vendors (AOR 2.73. 1.22-6.08) after adjusting for vendor-training on good milk handling practices (Table 7). Of the 152 vendors, only 6 (3.9%) male vendors had been trained on good milk handling practices of which 5 (83.3%) were found to be selling milk of poor compositional quality.

**Table 7:** factors associated with poor compositional quality of informally marketed raw cow milk by vendors in Lamu West Sub County, 2015

Characteristics	Poor Quality n (%)	Good Quality n (%)	Crude OR (95%CI)	p-value	Adjusted OR (95%CI)	p-value
**Age Group**						
≥ 45 years	23 (22.6)	79 (77.4)	0.68 (0.32-1.46)	0.326	-	-
< 45 years	15 (30.0)	35 (70.0)				
**Level of education**			1.00			
< Secondary Education	27 (23.7)	87 (76.3)	0.76 (0.33-1.74)	0.522	-	-
≥ Secondary Education	11 (30.0)	27 (70.0)	1.00			
**Sex**						
Male	21 (41.2)	30 (58.8)	**3.46 (1.61-7.47)**	**0.002**	**2.73 (1.22-6.08)**	**0.014**
Female	17 (16.8)	84 (83.2)	1.00			
**Trained on good milk handling practices**						
Trained	5 (83.3)	1 (16.7)	**17.12 (1.93-151.7)**	**0.004**	9.05 (0.97-84.3)	0.053
Not trained	33 (22.6)	113 (77.4)	1.00			
**Type of trade**						
Kiosk/shop	0 (0)	8 (100)	0.00	-	-	-
Hawker	38(26.4)	106 (73.6)				

## Discussion

This is the first study in the northern coastal Kenya to assess the compositional quality and milk safety in regard to presence of antimicrobial residues. Our results demonstrated that consumers of marketed row cow milk in this region were at risk of being exposed to public health problems associated with presence of antimicrobial residues in food of animal origin and consumption of adulterated milk. This study identified factors associated with the occurrence of the residues and the milk adulteration, and observed use of a herbal substance by both farmers and vendors in preservation of milk.

This study identified water as the main adulterant, which has also been identified by other studies elsewhere as the most common adulterant in the milk industry [17]. Water lowers the nutritional value of the milk, interferes with processing qualities of milk and poses a risk of contaminating the milk. Adulteration of milk by addition of water can easily be detected in the field using a lactometer [18].Other substances have been reported as milk adulterants, such as: chlorine, antibiotics, non-milk proteins, low value milk, milk powder, colour, preservatives, urea, liquid whey and water [17,19,20]. In north eastern Brazil, 41.2% of goat milk presented to the market was found to contain bovine milk [21]. A by-product from the cottage cheese industry called liquid whey has been reported to be used as a milk adulterant to increase the volume of milk after extracting proteins and fat [22]. Because of the wide variety of adulterants reportedly used in the dairy industry with diverse effects, there is need for routine monitoring of the milk market value chain right from farm level to assure food safety to consumers.

Findings of this study were higher than those of a study done in Nakuru, Narok, Nairobi and Kiambu counties of Kenya by Omore et al (2002) where 4.7% of milk specimens from household farms and 10.4% from marketing agents were found to be adulterated by addition of water [23]. The higher prevalence observed in our study can be associated with the young dairy industry in Lamu County where most of the produced milk is marketed raw directly to consumers as compared to Nakuru, Narok and Kiambu where bulk of the produced milk is sold to milk processing companies who are very strict on the quality of milk purchased. Milk of poor compositional quality is usually rejected by processors resulting in huge economic losses to farmers [2,24,25]. Milk processors also carry out extension services to farmers promoting good milking practices, a service lacking in Lamu County as there are no local milk processors.

This study demonstrated the occurrence of antimicrobial residues in marketed raw cow milk indicating that consumers are likely to be exposed to antimicrobial residues above EU MRLs each time they consumed the milk. The presence of antimicrobial residues in food is of concern as it contributes to development of drug resistance of human pathogens, allergic reactions and interference with growth of starter cultures in the milk processing industry [6,26,27]. The observed prevalence of residues in our study indicates a need to begin to address the problem both at the farm and market levels. This can be done through raising awareness amongst policy makers and implementers, farmers, vendors and consumers through specific extension messages [8]. A study in 1994 [28] conducted in Kiambu detected no residues in milk being supplied to milk cooperative societies in the county and was attributed to the high level of awareness and strict adherence to the withdrawal periods by farmers. This is consistent with findings in our study, where farmers who were aware of the danger of consuming milk with antimicrobial residues and those who had been trained on good milking practices were less likely to sell milk with antimicrobial residues.

This study observed higher prevalence of antimicrobial residues in milk marketed by farmers and vendors; compared to a study in 2005 [8] conducted in Nairobi, Nakuru and Narok, Kenya, where a prevalence of 11.1% amongst milk vendors and 16% amongst farmers was observed. The higher prevalence of antimicrobial residues observed in our study was attributed to lower levels of awareness of withdrawal periods amongst farmers [28].

Antimicrobial residue occurrence in milk has been reported globally. However, in countries with effective quality assurance systems, reports of residues in foods destined for the market are minimal or non-existent [8]. For example, in Brazil, a study to assess hazards in unpasteurized marketed milk at farm level found a prevalence of antimicrobial residues of 11.5% [29]. A study in the peri-urban areas of Accra and Kumasi cities in Ghana [14] found that 35.5% of samples of raw marketed milk, collected from different marketing agents including farmers, processors, wholesalers and retailers, were contaminated with antimicrobial residues. In Tanzania, a prevalence of 36% was observed in a study to investigate the risk of exposure to antimicrobial residues present in marketed raw milk in Mwanza and Dar es Salaam [30]. With such high prevalence observations in various countries, there is need to intensify safety assurance efforts both at farm and market levels, promote prudent use of antibiotics and observance of drug withdrawal period.

This study noted the existence of a non-antibiotic heat-sensitive inhibitor [16] in milk from Witu and Mpeketoni towns. From the interview findings, locals acknowledged to be using a herbal substance they called mpingo to preserve milk. This practice of using natural antimicrobials in milk preservation has been reported elsewhere [31-33]. Such substances are likely to affect growth of starter cultures in the milk industry, if the milk is not properly heated before start of processing. Little is known about the mpingo herb, which could have side effects to consumers.

In interpreting the findings of this study, it should be noted that sampling of farmers was independent from sampling of vendors. This study could not follow milk along the market value chain, that is, from individual farms to individual vendors, to determine the source or point of adulteration or the antimicrobial residues in the milk.

## Conclusion

This study identified the occurrence of antimicrobial residues above the set limits (EU MRLs) and adulteration of marketed raw cow milk through addition of water in Lamu West Sub-county. The antibiotics detected in the milk pose a health risk to the consumers by eliciting harmful effects. There is need to routinely test marketed milk, intensify public health education regarding milking and good milk handling practices, train farmers on strict adherence to antimicrobial use and withdrawal periods and impose stiffer penalties on those adulterating milk.

### What is known about this topic

- The compositional quality and prevalence of antimicrobial residues in central Kenya and areas surrounding Nairobi city are well known courtesy of several studies (2002, 2005), regular checks by the regulatory body, Kenya Dairy Board and multiple milk processing companies.

### What this study adds

- This study quantifies the extent of compositional quality and prevalence of antimicrobial residues in raw marketed cow milk in the coastal Kenya region, particularly Lamu West Sub County where there are no records of previous studies done in this region on the same topic;
- This study epitomizes the importance of raising awareness on good milking and milk handling practices amongst farmers and vendors, for good quality and safe milk.

## Competing interests

The authors declare no competing interest.
